# Wing morphometrics of *Aedes* (*Ochlerotatus*) *albifasciatus* (Macquart, 1838) (Diptera: Culicidae) from different climatic regions of Argentina

**DOI:** 10.1186/s13071-018-2888-3

**Published:** 2018-05-16

**Authors:** Maximiliano J. Garzón, Nicolás Schweigmann

**Affiliations:** 1 Departamento de Ecología, Genética y Evolución, Grupo de Estudio de Mosquitos, Universidad de Buenos Aires, Facultad de Ciencias Exactas y Naturales, Buenos Aires, Argentina; 2 Universidad de Buenos Aires, Consejo Nacional de Investigaciones Científicas y Técnicas, Instituto de Ecología, Genética y Evolución de Buenos Aires (IEGEBA), Facultad de Ciencias Exactas y Naturales, Buenos Aires, Argentina

**Keywords:** *Aedes albifasciatus*, Geometric morphometrics, Wings, Size, Shape, Dimorphism, Thermal ranges

## Abstract

**Background:**

Gene flow restrictions between populations of *Aedes albifasciatus*, the vector of Western equine encephalitis and *Dirophilaria immitis*, have been described in the central region of Argentina. Genetic and eco-physiological variations usually result in local forms reflecting the climatic regions. Mosquito wings and their different parts have ecological functions in flight and communication. Therefore, wing shape could be considered an aspect of sexual dimorphism, and its eco-physiological responses can be expressed as morphological changes induced by the environment.

**Methods:**

To compare the geographical and sexual variations with respect to wing shape and size in two *Ae. albifasciatus* populations from contrasting climates of Argentina (temperate: Buenos Aires, and the arid steppe of Patagonia: Sarmiento), the wings of adults reared in thermal trays at different constant temperatures (10–29 °C) were analyzed.

**Results:**

The wing size of *Ae. albifasciatus* showed inverse linear relationships with the rearing thermal condition and higher slope for Buenos Aires. In the cool range (10–17 °C), geographical size variations responded to the converse Bergmann’s rule, where Buenos Aires individuals were larger than those from Sarmiento. Sexual shape dimorphism occurred in both populations while geographical variation in shape was observed in both sexes.

**Conclusions:**

Buenos Aires individuals showed greater response sensitivity with respect to the size-temperature relation than those from Sarmiento. The converse Bergmann’s rule in size variation could be due to a higher development rate in Sarmiento to produce more cohorts in the limited favorable season. The shape could be more relevant with respect to the size in the study of population structures due to the size being more liable to vary due to changes in the environment. The geographical variations with respect to morphology could be favored by the isolation between populations and adaptations to the environmental conditions. Our results demonstrate that the shape and size of wing provide useful phenotypic information for studies related to sexual and environmental adaptations.

## Background

*Aedes* (*Ochlerotatus*) *albifasciatus* is a sylvatic mosquito from the Southern Cone of South America, with explosive abundances due to flood waters related to rainfall, overflow of rivers [1], and/or increase in the groundwater layers. *Aedes albifasciatus* was the first mosquito species incriminated as a vector of the western equine encephalitis virus [2]. This species has also been related to Saint Louis encephalitis virus [3] and Bunyamwera virus [4], and is a potential vector of *Dirofilaria immitis* [5]. This species is distributed from 17°S [6] to 54°S in Tierra de Fuego, Argentina [7]. Its geographical range includes different regions [8, 9] with different types of landscapes, environments and climates. *Aedes albifasciatus* has even been found to be adapted to extreme places such as elevated sites [10] and brackish water microenvironments [11], as well as tolerating strong wind bursts [12].

Previous studies have demonstrated that differences in the genetic divergence in *Ae. albifasciatus* are related to gene flow restrictions between breeding areas of different geographical populations [13, 14]. Otherwise, some temperature-dependent life-cycle parameters, such as development time and survival, present geographical variations [15]. These genetic and eco-physiological variations may result in local morphological forms (relative to size and shape) in response to contrasting climatic regions [16, 17].

Insect wings have ecologically important functions in flight and communication, and in fact various insects show intraspecific polymorphism in wing shape, making wings a target of natural selection [18]. The morphological characteristics of the different parts of the wing are closely related to the flight aerodynamics [19, 20].

In mosquitoes, only females are hematophagous and pathogen-competent and use their wings to ensure an accurate approach to other animals to suck their blood, while males can copulate with several mates and use wing beats to attract the opposite sex during courtship [21]. Therefore in mosquitoes, the wing shape and size could be considered an aspect of sexual dimorphism by their sex-specific function. Additionally, their eco-physiological responses include phenotypic plasticity, which can be expressed as changes in the morphology of the individuals of a population, such as those observed in the wings of mosquitoes. Plasticity can be induced by any environmental factor, and the resulting changes can vary from weak forms to well-adapted phenotypes [22]. The phenotypic variations related to the size and shape can be analyzed by morphometric techniques [23]. Both molecular and morphometric techniques [24, 25] have allowed the observation of intraspecific variability in insects, particularly in mosquitoes [26–28]. Geometric morphometrics has been used as a tool to analyze population structure [29], evolutionary units [30], genetic divergences in local populations [31], altitudinal variations [32] and different selective effects such as rearing conditions [33–35].

The objective of this study was to compare geographical and sexual morphological variations (wing shape and size) in two Argentinian populations of *Ae. albifasciatus* from contrasting climates and their response to different rearing temperature conditions. For this purpose, geometric morphometrics was used as a first approach to the comparative study at the population level.

## Methods

### Study sites

**Fig. 1:**
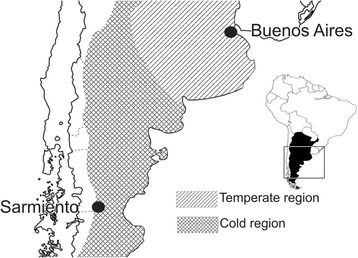
Study sites and climatic regions of Argentina (by Peel et al., 2007) where females of *Aedes* (*Ochlerotatus*) *albifasciatus* were captured

*Aedes albifasciatus* mosquitoes were collected in two sampling sites: Buenos Aires (34°36'S, 58°26'W, located in the Pampas plain) and Sarmiento Valley (45°35'S, 69°05'W, located in the Patagonian steppe) (Fig. 1). The climate in Buenos Aires is humid temperate [36], with warm summers (average 25 °C) and cool winters (average 12 °C).Winter is the wettest season of the year, with averages of 74–79% Relative Humidity (RH). In summer, the RH reaches values of 63–68%. The climate in Sarmiento valley is arid [36] with warm summers (average 18 °C), with daily desert thermal amplitude, and very cold winters (average 4 °C). In the cold season, the average monthly RH is approximately 75–80% (rainfall period), whereas in summer, the RH is 40% and precipitation does not reach the average of 15 mm. Moreover, the summer season is characterized by being very windy, with strong gusts (mean speeds ranging between 8 and 30 km/h) between calm periods [37, 38].

### Biological material

In the summer period (2010–2011), females of *Ae. albifasciatus* were caught in the field, taken to the laboratory (located near the natural breeding sites), and placed in three cages. For this species has not been possible to maintain colonies under laboratory conditions, probably due to its eurygamic characteristics, as in other species of the subgenus *Ochlerotatus* [39]. Consequently, to keep 150 individuals per cages approximately, dead individuals were replaced daily (about 30–50 adults). As a source of blood for egg laying, females were blood-fed on mammals (rabbits or nursing mice), where laboratory animal ethics protocols were followed according to the University of Buenos Aires regulations (R 4081/2004).

A Petri dish with absorbent paper on damp cotton was offered as an oviposition substrate in each of the cages with females. For the experiments, different portions of these substrates were randomly selected for the purpose of using eggs from different individuals. Eggs were induced to hatch by immersing them in a yeast solution. Then, approximately 30 larvae were placed randomly in individual containers (cylindrical containers measuring 3 cm in diameter and 5 cm high with 10 ml of dechlorinated tap water) in thermal trays at constant temperatures in a range of 10–29 °C (3° C step) until they reached their adult form [15]. As the temperatures of the trays could be affected by ambient conditions, we placed temperature data loggers (Hobo®, Onset Computer Corporation, Bourne, MA, USA) in the trays, and took the mean recorded temperature instead of the system thermostat sensor reading. The photoperiod during rearing was 14:10 h (light: dark) and the individuals were fed daily with an aliquot of dog chow (Purina®, San Luis, MO, USA), as follows: instar I: 0.2 mg/day, instar II: 0.3 mg/day, instar III: 0.4 mg/day and instar IV: 0.6 mg/day [40]. After completion of development, adults which emerged successfully were sacrificed at freezing temperatures (-12 °C). A total of 135 specimens were able to be photographed and analyzed: 24 males and 36 females from Buenos Aires and 32 males and 43 females from Sarmiento valley.

To evaluate the geographical variation and sexual dimorphism as a function of the thermal range, the temperatures tested were divided into two sub-ranges: cool (10–17 °C) and warm (19–29 °C), based on the fact that the populations studied respond differently to the time of development as a function of warm or cool temperatures [15], which directly affects their size.

### Wing processing, centroid size and shape

**Fig. 2:**
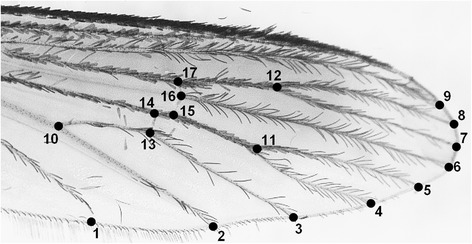
Location of the 17 landmarks (LMs) on the wings of *Aedes* (*Ochlerotatus*) *albifasciatus*

The left wings of each individual were removed from the thorax by using a fine clamp, placed between glass slides and photographed with a camera (Leica® DFC 295; Leica Camera AG, Solms, Germany) coupled with a stereoscopic microscope (Leica® S8 APO; Leica Camera AG, Solms, Germany). Once the digital image was obtained, 17 points of the wing (natural intersections given by the venation) or landmarks (LMs) (Fig. 2) were selected from references, and the software tps-DIG 2.16 [41] was used to generate Cartesian coordinates in two dimensions for each individual. The LM configurations obtained were transferred, rotated and scaled according to the generalized Procrustes method [42], using MorphoJ® software 1.05 [43]. Thus, the new Procrustes coordinates were generated to be used as shape variables. To compare the size of the wing between the different groups (sex or population), the centroid size (CS), derived from Cartesian coordinate data, was used as an isometric size estimator. The CS is defined as the square root of the sum of the square of the distances between the center of the configuration of the LM (or centroid) and each LM [42]. Since the CS is based on Cartesian coordinates (XY), the result of the calculation mentioned above is a one-dimensional scale. For the analysis, the morpho-geometric sizes (CS) were logarithmized (log CS).

The software used to obtain, process and analyze the data (tps-DIG 2.16, MorphoJ®) is freeware (http://life.bio.sunysb.edu/morph/).

### Data analysis

To study the relationship between size and temperature, the logarithm of the CS against the rearing temperature was linearly fitted for each sex and population separately. Then, the slopes were compared by a test of parallelism in both populations and sexes [44]. The size differences between sex and population were statistically evaluated using the log CS in a parametric two-way ANOVA [45]. Due to the low survival of the individuals in both populations at warm range, only data for the cool range were subjected to statistical tests.

The allometric influence of wing size on wing shape was assessed by multivariate regression of the Procrustes coordinates (shape) against CS, using a permutation test with 10,000 randomizations [46, 47].

Two-way multivariate analysis of variance (MANOVA) was used to compare and discriminate wing shapes between the populations and sexes. Previously, the variables of the shape (without the allometric effect) were summarized by means of a principal components analysis (PCA) where the PCs generated were taken as new variables of the shape. Canonical variate analysis (CVA) combined with discriminant analysis (DA) was performed [47] to evaluate the differences between sex and population.

To compare Procrustes distances between groups, a permutation test with 10,000 iteration rounds was performed. The square root of the sum of the squared distances between the corresponding LMs of two aligned configurations is an approximation of the Procrustes distance [48], which is a measure of shape variation. In all cases, a significance level α = 0.05 was used. To visualize the morphological changes in the wings, deformation vectors were used with respect to a consensus configuration and a thin-plate spline or wireframe scheme [43, 47], using the software MorphoJ® 1.05. The comparison between thermal ranges respect to the shape was not analyzed due to a low sample size in both populations at warm range.

The variability of population and sex shapes was estimated with CS of scatter plots within the morpho-space formed by the first PCs (for instance, PC1:X and PC2: Y), similar to that described in Louise et al. [49]. For the comparison between groups, we generated CS values and performed two-way ANOVA.

## Results

### Centroid size

**Fig. 3:**
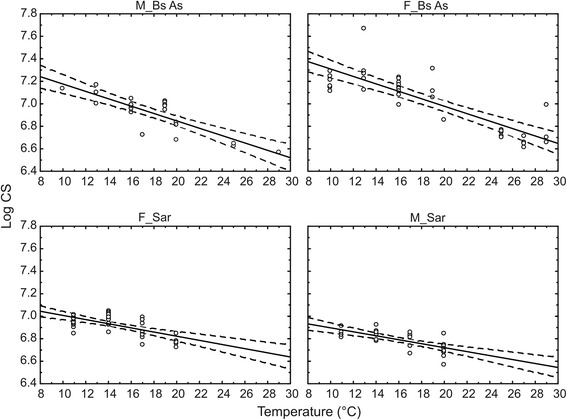
Wing size [Log (centroid size)] as a function of the rearing temperature. The solid lines indicate the linear fit and the dotted lines the confidence level

For both populations (Buenos Aires, Sarmiento) and for both sexes (males and females), the wing size (log CS) showed an inverse relationship with the rearing temperature (Fig. 3). The simple linear regressions were significant for the slope in all the cases, and the linear fit (*r*^2^) was better for the individuals from Buenos Aires (males Buenos Aires: *r*^2^ = 0.7110, *P* = 0.0000004, y = 7.5032 - 0.0328*x; females Buenos Aires: *r*^2^ = 0.7076, *P* = 0.00003, y = 7.6401 - 0.0331*x; males Sarmiento: *r*^2^ = 0.5097, *P* = 0.000004, y = 7.0732 - 0.0176*x; and females Sarmiento: *r*^2^ = 0.4168, *P* = 0.000003, y = 7.1921 - 0.0185*x). Tests of parallelism indicated no differences between sexes for each population (Buenos Aires: *n* = 60, *F*_(1, 56)_ = 0.00054, *P* = 0.9816; Sarmiento: *n* = 75, *F*_(1, 71)_ = 0.04, *P* = 0.8433), but there were differences between populations within each sex (males: *n* = 56, *F*_(1, 51)_ = 8.16, *P* = 0.0062; females: *n* = 79, *F*_(1, 75)_ = 5.86, *P* = 0.0179).

**Fig. 4:**
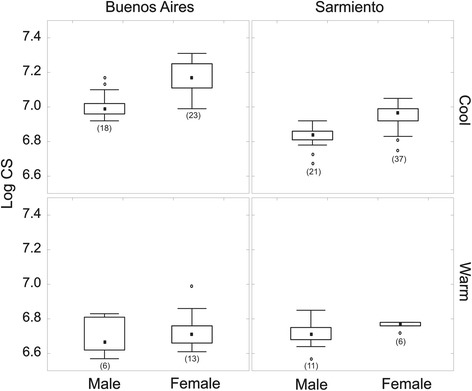
Centroid size (Log CS) distribution in the wings of *Aedes* (*Ochlerotatus*) *albifasciatus* populations from Buenos Aires and Sarmiento, for cool (10–17 °C) and warm (19–29 °C) thermal ranges. Numbers in parentheses indicate sample sizes (*n*)

The mean sizes (log CS) in the cool thermal range were: males Buenos Aires (*n* = 18) = 7, males Sarmiento (*n* = 21) = 6.83, females Buenos Aires (*n* = 23) = 7.2 and females Sarmiento (*n* = 37) = 6.95. The ANOVA showed no interaction between sex and population (*F*_(1, 93)_ = 3.35, *P* = 0.0704). Regarding the main effects, the results showed sexual size dimorphism (*F*_(1, 93)_ = 91.36, *P* < 0.0001), with females being larger than males regardless of the population and with the individuals from Buenos Aires being larger than those from Sarmiento (*F*_(1, 93)_ = 159.58, *P* < 0.0001) regardless of the sex (Fig. 4).

The size means in the warm thermal range were: males Buenos Aires (*n* = 6) = 6.69; males Sarmiento (*n* = 11) = 6.71; females Buenos Aires (*n* = 13) = 6.73 and females Sarmiento (*n* = 6) = 6.77 (Fig. 4).

### Shape

The allometric test was significant (*P* = 0.001) and the proportion of total shape variation explained by the fact that the regression (analog to *r*^2^) was lower than 9%. Although the allometric level was low, it was removed for the shape analyses.

After removing the allometric effect, the PCA was carried out on shape variables (regression residuals), where the first six components (explaining 81% of the variance) were conserved according to the Kaiser criteria (eigenvalues greater than 1) as new shape variables. MANOVA showed that the shape was significantly different between populations and between sexes (Pillai, Lawley-Hotelling and Roy tests with: *F*_(6, 124)_ = 186.12 and *P* < 0.0001 for sex level; *F*_(6,124)_ = 13.83 and *P* < 0.0001 for population level; and *F*_(6,124)_ = 1.57 and *P* = 0.1401 for the sex-population interaction).

**Fig. 5:**
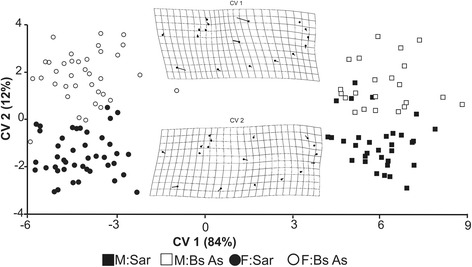
Canonical variables (CV1 and CV2) for wing shape configuration of *Aedes* (*Ochlerotatus*) *albifasciatus*: Males from Sarmiento (filled squares) and Buenos Aires (empty squares); Females from Sarmiento (filled circles) and Buenos Aires (empty circles). Changes in wing shape through the canonical variables (CV1: 84% and CV2: 12%) are illustrated with a thin-plate spline. Thick points indicate the consensus configuration and the vectors (segments) indicate the relative relevance landmarks in the discrimination

The CVA revealed differences in shape between sexes and populations (Fig. 5). Canonical variable 1 (CV1), which explained 84% of the total variance, showed a clear sex separation, whereas CV2, which explained 12% of the total variance, indicated a distinction between populations. The Procrustes distances of shapes between sexes (Buenos Aires_males-females_: 0.89; Sarmiento_males-females_: 0.88) were higher than between populations (males_BuenosAires-Sarmiento_: 0.027; females_BuenosAires-Sarmiento_: 0.028). The permutation test (10,000 iteration rounds) for Procrustes distances was significant (*P* < 0.005).

**Fig. 6:**
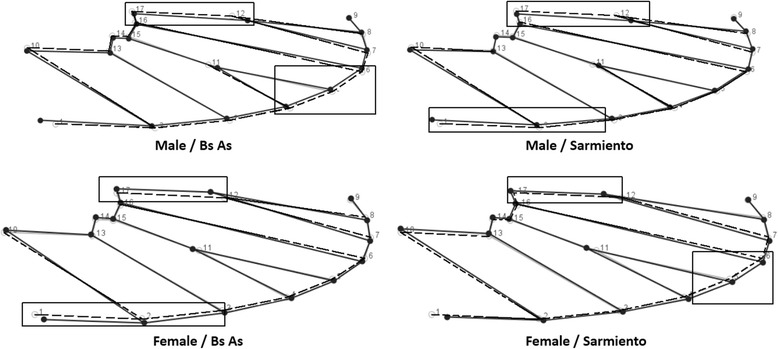
Wireframe scheme for the average wing shape (dotted lines) of *Aedes* (*Ochlerotatus*) *albifasciatus* and the tendency of the variation (continuous line) by populations and sexes. The rectangles indicate zones of differences with respect to the average shape

These differences between sexes are shown in Fig. 6, where rectangles indicate the relevant area of the wing. The differences were at the wing width level and, as expected, these were more contrasting between the sexes, where the narrower form was that for males. Regarding the differences between populations, although less conspicuous, the results show that the wings of Sarmiento individuals are slightly thinner with respect to Buenos Aires (Fig. 6).

The thin-plate spline showed that the LMs 1, 11 and 12 were the most important in the shape changes, associated with CV1, in the sex separation. In CV2, LMs 1, 2, 10 and 9 explained the remaining variance between the populations.

The DA of pairs allowed separating the populations of the same sex and the sexes within the same population. Because the sample size was relatively small in relation to the variables to be analyzed (17 LMs), the cross-validation classification was carried out (Table 1). In females, 25% of the specimens from Buenos Aires were misclassified as Sarmiento and 21% of those from Sarmiento were classified as Buenos Aires, whereas in males, 33% of the specimens from Buenos Aires were misclassified as Sarmiento and 28% of those from Sarmiento were misclassified as Buenos Aires. No classification errors were detected between sexes (Table 1).

**Table 1:** Cross-validation classification for *Ae. albifasciatus* from discriminant analysis of pairs (by populations and sexes)

Proportion of misclassification				
Groups compared	BsAs F	Sar F	BsAs M	Sar M
Geographical variation				
BsAs F - Sar F	9/36	9/43		
BsAs M - Sar M			8/24	9/32
Sexual dimorphism				
BsAs F- BsAs M	0/36		0/24	
Sar F - Sar M		0/43		0/32

*Abbreviations*: *BsAs F* Buenos Aires females, *BsAs M* Buenos Aires males, *Sar F* Sarmiento females, *Sar M* Sarmiento males

### Shape variability

Five CS values were obtained for shape variability in each group. ANOVA showed that the shape variability of females (mean = 558.7) was significantly higher than that of males (mean = 393.6) (sex main effect: *F*_(1, 16)_ = 40.71, *P* = 0.0001). No significant differences were observed between populations (population main effect: *F*_(1, 16)_ = 0.65, *P* = 0.4315). The sex-population interaction was not significant (*F*_(1, 16)_ = 1.82, *P* = 0.1959).

## Discussion

The geometric morphometrics of the wings for *Ae. albifasciatus* allowed us to comparatively study the sexual dimorphism and the variability of individuals from different regions. It is known that geographical differences may exist in the vector competence in mosquitoes, observed in differences in infection and transmission rates [50, 51].

A negative linear relation between the body size magnitude and the breeding temperature has been observed for wing lengths [52, 53], femur lengths [53], cephalic capsule [54] and dry weight [52]. Although there is a good fit between wing length and centroid size [55], the centroid size is more representative of the magnitude of the individual because its multidimensionality summarizes the global size variation [23]. The slopes indicate that the response of size change (centroid) to the temperatures for both sexes are the same. This would indicate the same metabolic response, where the physical assimilation of nutrients is lower with the increase in temperature, since the development is more accelerated [15, 56].

### Sexual size and shape dimorphism

In the cool thermal range, there was sexual dimorphism of sizes for both populations. This difference is the result of a lower development time for males (protandry) resulting in a smaller body size [57]. In most insect species, females are expected to reach a larger size than males due to the important role of population fitness [58]. In the warm thermal range a tendency of sexual dimorphism with larger females is observed (small size of the sample did not allow applying a statistical test).

The results suggest a sexual shape dimorphism for *Ae. albifasciatus* populations, with more marked differences than between the geographical populations. This sexual dimorphism was evident, resulting in accurate classifications (100% success) (Table).

The wings of the females were wider in the anterior-posterior sense, while the males had narrower wings. The wireframe (Fig. 6) confirmed this difference. This feature could optimize flight according to its sex-specific functionality, since *Ae. albifasciatus* is eurygamic, namely, it mates in swarms in open spaces [59] and, as in other species, each sex would recognize its mating pair through the sound of its wings during flight [60]. On the other hand, females must fly accurately to find the host [61] and then find a suitable site for oviposition. The sexual shape dimorphism would represent another evolutionary example in homologous structures [62], where the shape is canalized by sexual selection or other evolutionary mechanisms [21].

Regarding shape variability, the wings of *Ae. albifasciatus* females showed greater variability of shapes than those of males, indicating a greater sensitivity to the different rearing temperature conditions [58] and suggesting a more conspicuous phenotypic plasticity. Something similar has been recorded for females of *Ae. albopictus*, in which the rate of change was higher than that recorded for males [61].

### Geographical variation in size and shape

The centroids size-temperature relation in *Ae. albifasciatus* individuals from Sarmiento showed a lower slope than those from Buenos Aires, suggesting a more robust or less sensitive response to the gradual change in the rearing temperature. This characteristic could be a local adaptation to the local climatic region (Patagonian) where the larval state must resist the daily thermal amplitude [36]. Local adaptations have also been suggested for other life-cycle parameters such as development time and survival [15].

Insects usually respond to environmental conditions following the James rule [63] (which is similar to Bergmann’s rule [64]) or the converse Bergmann’s rule [65]. The James rule explains the increase in body size with latitude or decreasing temperature [66]. In contrast, in the converse Bergmann’s rule, the size decreases with latitude, as a consequence of a shorter favorable season and an increase in developmental metabolism. The latter rule is common among univoltine insects [67] and has a genetic basis since this type of response has been observed both in field and laboratory studies [68]. Under cool rearing thermal ranges in the laboratory, *Ae. albifasciatus* individuals from Buenos Aires were larger than those from Sarmiento (Fig. 4). The lower accumulation of biomass by the individuals would be due to a higher rate of development [15] as a physiological response to the local environmental conditions [69]. Therefore, the individuals from Patagonia could follow the converse Bergmann’s rule, although *Ae. albifasciatus* is a multivoltine species, where an increase in metabolism could produce more cohorts in a limited favorable season (summer) [12], resulting in individuals of smaller size than those from Buenos Aires.

In Buenos Aires and other temperate populations, individuals rear and can be active throughout the year [70, 71], suggesting a lower selective pressure (accelerated metabolism) with respect to the development time observed in the Sarmiento population.

The geographical differences of wing shape in *Ae. albifasciatus* could indicate a geographical polymorphism or population structure, favored by the isolation between populations, since Sarmiento valley is surrounded by a desert steppe matrix. On the other hand, the shape differences between populations should reflect the arid or temperate environmental conditions [24].

The difference of the wing shape between studied populations could indicate an adaptation in relation to flight dynamics [19, 20]. In a windy environment such as the Patagonian arid steppe during the summer time [38], the favorable *Ae. albifasiatus* season [12], thinner wings could be more favorable to avoid being displaced.

It is known that the individual or population variations of the wing shape and size are selected depending on the characteristics of the environment [72]. The architecture of morphological characters responds to the engagement between the demands of the environment and those of the genome [25]. Therefore, complementary molecular studies could explain the proportion explained by the genotype and the proportion explained by the environment.

In many circumstances, alternative phenotypes in response to environmental changes could be an adaptive strategy to maximize fitness in variable environments [73, 74].

## Conclusions

The results of the present study evidenced sexual shape dimorphism in *Ae. albifasciatus* for the two populations and sexual size dimorphism in cool temperature rearing conditions (and probably a warm range). Shape geographical variation in individuals of *Ae. albifasciatus*, regardless of the sex, could indicate a population polymorphism between geographical regions. Size geographical variation was observed only in cool rearing temperatures, according to the converse Bergmann’s rule. Furthermore, an inverse relationship between size and temperature was demonstrated for the two populations studied, although individuals from Buenos Aires showed greater response sensitivity than those from Sarmiento. Our results demonstrate that the shape and size of wing provide useful phenotypic information for studies related to sexual and environmental adaptations.

## Abbreviations

RH: Relative humidity
LMs: Landmarks
LM: Landmark
CS: Centroid size
ANOVA: Analysis of variance
MANOVA: Multivariate analysis of variance
PCA: Principal components analysis
CVA: Canonical variate analysis
DA: Discriminant analysis
PC: Principal component
CV: Canonical variate
